# Age-Friendly and Dementia-Friendly Massachusetts

**DOI:** 10.1093/geroni/igaf122.350

**Published:** 2025-12-31

**Authors:** James Fuccione

**Affiliations:** Massachusetts Healthy Aging Collaborative, Boston, Massachusetts, United States

## Abstract

The Massachusetts Healthy Aging Collaborative (MHAC), funded by Point32Health Foundation, is a statewide, cross-sector network committed to advancing inclusive healthy aging and age- and dementia friendly communities throughout the state. There are now 131 active age-friendly and 122 active dementia-friendly communities with 81 involved in both initiatives. In 2018, MA became the second US state to become age-friendly through AARP’s Network of Age-Friendly States and Communities and the first with an action plan. MHAC supported the newly-rebranded Executive Office of Aging & Independence on a community engagement process to refresh this plan that will be launched with a range of partners and communities in 2025. MHAC provides an age-friendly voice as part of multiple state commissions and workgroups that promote housing, digital equity, safe pedestrian and bicycle infrastructure, falls prevention and dementia-friendly community design. MHAC has supported equity in the movement with its “Healthy Aging for All Guide” and co-leads a $3.3 million digital equity initiative that supports older adults in 74 cities and towns. MHAC uses data to inform all of it’s outreach and advocacy work and helps communities to leverage local data as well.

